# Associations between Dietary Inflammatory Index Scores and Inflammatory Biomarkers among Older Adults in the Lothian Birth Cohort 1936 Study

**DOI:** 10.1007/s12603-019-1221-y

**Published:** 2019-06-24

**Authors:** Janie Corley, N. Shivappa, J. R. Hébert, J. M. Starr, I. J. Deary

**Affiliations:** 1 Department of Psychology, University of Edinburgh, Edinburgh, EH8 9JZ UK; 2 Centre for Cognitive Ageing and Cognitive Epidemiology, Department of Psychology, University of Edinburgh, 7 George Square, Edinburgh, EH8 9JZ, Scotland UK; 3 Royal Victoria Building, Western General Hospital, Porterfield Road, Edinburgh, UK; 4 Cancer Prevention and Control Program, University of South Carolina, Columbia, SC 29208 USA; 5 Department of Epidemiology and Biostatistics, Arnold School of Public Health, University of South Carolina, Columbia, SC 29208 USA; 6 Connecting Health Innovations, LLC, 1417 Gregg Street, Columbia, SC 29201 USA

**Keywords:** Dietary inflammatory index, inflammation, validation, C-reactive protein, interleukin-6

## Abstract

**Objectives:**

Chronic low-grade inflammation is a key underlying mechanism in several age-related chronic conditions and previous studies have shown that diet can modulate the inflammatory process. We investigated the ability of the Dietary Inflammatory Index (DII®), a summary measure of dietary inflammatory potential, to predict concentrations of plasma inflammatory markers in a sample of older people.

**Design:**

Cross-sectional and 3-year follow-up analysis of Lothian Birth Cohort 1936 (LBC1936) study data.

**Setting:**

Baseline data collection occurred between 2004 and 2007 in Edinburgh, Scotland. Participants: Men and women (n 928, age ∼70 at baseline) living in Edinburgh and surrounding regions who are surviving participants of the Scottish Mental Survey of 1947.

**Measurements:**

Energy-adjusted DII (E-DII) scores at age 70 (derived from a food-frequency questionnaire), plasma concentrations of inflammatory biomarkers at age 70 (C-reactive protein (CRP), fibrinogen) and age 73 (CRP, fibrinogen, hs-CRP, Interleukin-6 (IL-6)). Analyses were performed using multivariable logistic regression adjusting for age, sex, smoking, body mass index, physical activity, and hypercholesterolaemia.

**Results:**

Higher E-DII scores (pro-inflammatory diet) were associated with increased odds of elevated CRP (>3mg/L) at age 70 (OR 1.12; 95% CI: 1.02, 1.24, P = 0.02), and elevated IL-6 (>1.6pg/ml) at age 73 (OR 1.11; 95% CI: 1.00, 1.23, P = 0.04), but not with fibrinogen.

**Conclusion:**

These results are consistent with the ability of the DII to predict inflammatory biomarker concentrations and suggest that diet plays a role in the regulation of inflammation, even after controlling for potential confounders. This validation study provides support for using the DII in research among older populations.

## Introduction

Chronic inflammation, as indicated by the continuous presence of serum inflammatory mediators such as C-reactive protein (CRP), fibrinogen, and various interleukins, is a risk factor for numerous age-related, chronic disorders such as diabetes, cardiovascular disease (CVD), cancer, and the metabolic syndrome (1, 2), as well as increased frailty and cognitive decline (3). Reducing inflammation may help to prevent or treat these conditions. Chronic, low-grade inflammation, has a complex and multifocal etiology, and may be modified by endogenous and exogenous factors. Dietary factors have been consistently found to regulate inflammation (4–7). Healthy diets (e.g., the Mediterranean diet, rich in fruits and vegetables) typically have been associated with lower inflammation levels, whereas Western-style diets (e.g., high in fat and simple carbohydrate) have been associated with higher levels of inflammatory markers (8–11). Specific nutrients such as vitamins C, D, and E, beta-carotene, n3-PUFA, flavonoids, and other dietary components such as fibre and a moderate alcohol intake, have been associated with lower levels of inflammation (12, 13).

The Dietary Inflammatory Index (DII®) was developed to estimate the overall inflammatory potential of the diet on an anti- to a pro-inflammatory continuum (14). It is derived from the literature linking diet and inflammation, incorporating cell culture, animal, and human studies. The DII includes macronutrients, micronutrients, and other common dietary components such as flavonoids and caffeine. Previously, the DII has been shown to predict levels of inflammatory markers, including CRP and IL-6, in cross-sectional (15–21) and longitudinal studies (22). In the Seasonal Variation of Blood Cholesterol Study, higher DII scores were associated with values of hs-CRP >3 mg/l (OR 1.08; 95% CI 1.01, 1.16, P = 0.035 for the 24HR subset; and OR 1.10; 95% CI 1.02, 1.19, P = 0.015 for the 7-Day Dietary Recall) (16) corresponding to an 8% and 10% increase, respectively, in risk for each DII point (in a maximum range of about 18 points). In the Women’s Health Initiative, the DII was associated with four inflammatory biomarkers with beta estimates (95% CI) comparing the highest with lowest DII quintiles as follows: interleukin-6: 1.26 (1.15–1.38), P_trend_ < 0.0001; tumour necrosis factor alpha receptor 2: 81.43 (19.15–143.71), P_trend_ = 0.004; dichotomized hs-CRP (OR for higher vs. lower hs-CRP): 1.30 (0.97–1.67), P_trend_ = 0.34; and the combined inflammatory biomarker score: 0.26 (0.12–0.40), P_trend_ = 0.0001 (19). Also, the DII has been associated with a range of outcomes including several types of cancer (19, 23–28), cardiovascular disease (29, 30), metabolic syndrome (20, 31), and mortality (32–34). Specifically, in the UK, higher DII scores were associated with increased risk of recurrent depression among women, and all-cause mortality (35, 36).

Previous validation studies have been conducted among young and middle-aged adults in Europe and the US, but not among a British population, or in a sample of exclusively older adults. Older people tend to have higher levels of systemic indicators of chronic inflammation (37). This is often referred to as ‘inflammaging’, and is a significant risk factor for morbidity and mortality (38). A state of mild or low level inflammation is associated with, and predictive of, many aging phenotypes such as changes in body composition. Obesity is a condition that tends to increase in frequency with age (39) and can cause or contribute to inflammaging (40). However, the DII has not yet been well-studied in aging populations. It is unclear whether associations between the inflammatory potential of diet and low-grade systemic inflammation among older adults may differ from those observed in younger populations. Identifying pathways that control or ameliorate age-related inflammation is important in order to determine whether there are benefits to dietary modification, on reducing so-called ‘inflammaging’ and adverse health outcomes, at the population level.

In the present study, our objective was to conduct a validation study of food-frequency questionnaire (FFQ)-derived DII scores in an older UK sample, The Lothian Birth Cohort 1936, by evaluating its association with a several inflammatory biomarkers (CRP, hs-CRP, fibrinogen, and IL-6).

## Methods

### Participants

The Lothian Birth Cohort 1936 (LBC1936) study is a longitudinal study of the determinants of normal cognitive aging in Scotland, and has been described in detail elsewhere (41, 42). Briefly, a total of 1,091 relatively healthy men and women about 70 years of age (mean 69.5, sd 0.8), most of whom previously participated in the Scottish Mental Survey of 1947 at age 11, were recruited between 2004 and 2007. All data were gathered through interviews, neuropsychological testing, blood samples, clinical measurements, and questionnaires, by psychologists and medical staff. A total of 866 attended a 3-year follow-up assessment when they were about 73 years old (mean 72.5, sd 0.7). For the current study, we excluded ninety-seven participants at age 70, and sixty-nine participants at age 73, with CRP and hs-CRP values >10 mg/L to limit confounding due to acute infection (43). After exclusions and missing data, dietary data and inflammatory biomarker data were available for 928 participants at age 70, and 765 participants at age 73.

### Data collection

#### Dietary data

Dietary data were collected at baseline using the Scottish Collaborative Group 168-item Food Frequency Questionnaire (FFQ), version 7.0 (44, 45) reflecting average dietary intake over the previous two to three months. Participants were asked to indicate how often they consumed each food and drink item from a list of frequencies (7+ d/day; 4–6 d/day; 2–3 d/day; 1 d/day; 4–6 d/week; 2–3 d/week; 1 d/week; 1–3 d/month; rarely or never), according to a common unit or portion size. This semi-quantitative FFQ was developed for use in older adults. In previous studies, the repeatability and validity of the FFQ was demonstrated against 4-day weighed food diaries (46, 47). Dietary intake in later life was found to be reasonably stable in the short term, and the authors reported good validity for most nutrients in community-dwelling older populations. FFQ responses were used to calculate DII scores for all participants.

#### Dietary Inflammatory Index

The DII® is a score originally developed using 45 parameters (specific foods and nutrients) as indicated by a literature review of associations with six inflammatory biomarkers (IL-1β, IL-4, IL-6, IL-10, TNFα, and CRP) quantified in around 2000 peer-reviewed articles. An individual’s DII score, which is computed from the FFQ, is compared to food consumption data from eleven populations around the world. Details of the development (14) and construct validation (16) of the DII have been described elsewhere. The overall DII score characterises an individual’s diet on a continuum from anti-inflammatory (the low end of the scale) to pro-inflammatory (the high end of the scale). The DII score can be calculated using >20 items from the desired list of food parameters. In the LBC1936 study, 26 parameters were available for inclusion in the overall DII score, namely: energy; carbohydrate; protein; total fat; saturated fat; cholesterol; iron (which contribute positively to the calculation of the overall DII score), and polyunsaturated fat; fibre; thiamine; riboflavin; vitamins B6, B12, C, D, E; zinc; selenium; folic acid; beta-carotene; flavonol; flavonones; flavones; alcohol; tea; and total caffeine (which contribute negatively to the calculation of the overall DII score). The dietary parameter values were summed to create an overall DII score. To control for the effect of total energy intake, we calculated the energy-adjusted version of the DII per 1,000 calories of food consumed (the E-DII), using the energy-standardized version of the world database. All calculations in the current study were performed according to standard DII protocol (14).

#### Inflammation biomarkers

Blood samples were drawn from participants at age 70-wave 1 (baseline) and at a 3-year follow-up at age 73-Wave 2, by a qualified nurse, immediately centrifuged, and stored frozen at −80°C. Serum CRP (mg/l) and fibrinogen (g/l) concentrations were measured at ages 70 and 73. High-sensitivity CRP (hs-CRP mg/l) and IL-6 (pg/l) concentrations were measured at age 73 only.

The (non-hs) CRP assay was performed using a dry-slide immuno-rate method on OrthoFusion 5.1 F.S analysers (Ortho Clinical Diagnostics). The CRP assay method has low sensitivity in the lower range of CRP values; approximately 45% of participants were in the single lowest category (1.5 mg/l). The fibrinogen assay was performed using an automated Clauss assay (TOPS coagulometer; Instrumentation Laboratory, Warrington, UK). Hs-CRP and IL-6 were determined using high-sensitivity enzyme-linked immunosorbent assay kits (R&D Systems, Oxon, UK).

#### Covariates

Data including education (years of full-time formal schooling), smoking status (current, ex- or never), and history of disease (hypertension, CVD, hypercholesterolaemia, stroke, and diabetes) were collected at interview. Data on physical activity (number of days per average month doing any sport or physical exercise that makes you out of breath and sweat for >20 minutes at a time) and depression (derived from the Hospital Anxiety and Depression Scale, HADS-D; (48)) were collected using questionnaires. The HADS-D score ranges from 0 to 21 with lower scores indicating fewer depressive mood symptoms. Body mass index (BMI) was derived from height and weight measures taken by a nurse during the clinical assessment and calculated as kilograms per metre squared.

### Statistical analysis

All inflammation biomarkers were categorised according to conventional and clinically relevant cut-off points. In the main analyses, as in previous LBC1936 studies (49, 50) and other DII validation studies (e.g. 15–17, 19, 22), all CRP values were dichotomised at the level of 3 mg/l: normal (3 mg/l or less) and elevated (greater than 3 mg/l). The relevance of the 3 mg/l cut-off has been implicated in the prediction of CVD (43). Fibrinogen values were dichotomised at the level of 4.0 g/l: normal (4.0 g/l or less) and elevated (greater than 4.0 g/l) in line with the standard UK clinical reference range. IL-6 values were dichotomised at the detection level of 1.6 pg/ml: normal (1.6 pg/ml or less) and elevated (greater than 1.6 pg/ml). This cut-off level is consistent with a previous DII validation study (17).

For descriptive purposes, participants’ characteristics were summarised according to E-DII tertiles using frequencies (n (%)) for categorical variables and means ± standard deviations for continuous variables. Comparison of participants’ characteristics across the E-DII tertiles were made using Chi-square tests for categorical variables and ANOVA for continuous variables. Normality of the distribution was assessed using Q-Q plots and the Kolmogorov-Smirnov test.

The associations between E-DII scores (as a continuous variable) and each inflammation biomarker available at age 70 and age 73 were analysed using multivariable logistic regression models. Model 1 was fit for each inflammation biomarker as the dependent variable and a continuous measure of the E-DII as the predictor variable, adjusting for age (exact age in days at the time of testing) and sex. Model 2 adjusted for multiple covariates, namely, age, sex, smoking status (current, ex, never), body mass index, physical activity, and hypercholesterolaemia. Models were not adjusted for total energy intake because it is one of the DII components and was accounted for in the formulation of the E-DII scores. All analyses were performed using SPSS software (version 23). A P-value of < 0.05 was considered significant.

## Results

Table 1 presents the dietary parameters used to calculate the E-DII across the E-DII tertiles. Higher E-DII scores (indicating a more pro-inflammatory diet) were associated with a significantly higher intake of calories, carbohydrates, alcohol, cholesterol and saturated fat. Conversely, there were significant and linear inverse associations between the E-DII and intake of fibre, flavonoids, and various specific nutrients (thiamine, vitamins B6, C, D, E, iron, selenium, folic acid and beta-carotene). These associations were all in the expected direction for the DII, with the exception of iron. Non-significant associations were observed between the E-DII score and protein, riboflavin, vitamin B12, zinc, and total caffeine.

**Table 1:** Dietary parameters used in the calculation of the energy-adjusted Dietary Inflammatory Index (E-DII) across the tertiles of the E-DII at baseline (Mean scores and standard deviations)

		**Tertiles of Dietary Inflammatory Index (E-DII)**					
	**Tertile 1 (n 309)**	**Tertile 2 (n 310)**			**Tertile 3 (n 309)**		
**Dietary parameter**	**m**	**sd**	**m**	**sd**	**m**	**sd**	**P**
E-DII scores	−0.13	0.83	0.64	0.42	2.37	0.76	<0.001
Energy, kcal	7711.62	3109.30	8073.46	2714.42	8447.17	2703.82	0.006
Carbohydrates, g	108.78	49.26	115.06	45.53	123.94	52.05	0.001
Protein, g	76.21	28.59	75.19	25.41	74.74	26.91	0.79
Alcohol, g	8.518	10.05	12.91	18.83	14.51	19.29	<0.001
Fibre, g	19.74	10.58	15.74	5.92	13.23	5.26	<0.001
Cholesterol, mg	248.64	110.47	279.02	108.23	318.00	137.02	<0.001
Saturated fat, g	23.88	11.93	28.83	11.61	34.05	14.24	<0.001
PUFA, g	11.81	6.03	11.87	5.09	10.77	4.35	0.01
Thiamin, mg	1.62	0.67	1.52	0.56	1.46	0.57	0.004
Riboflavin, mg	1.95	0.81	1.98	0.90	1.94	0.80	0.84
Vitamin B6, mg	2.22	0.92	2.05	0.78	1.88	0.71	<0.001
Vitamin B12, µg	6.79	3.36	6.77	3.80	6.44	3.00	0.35
Vitamin C, mg	145.46	96.16	104.36	50.18	76.15	40.15	<0.001
Vitamin D, µg	4.39	2.76	4.23	3.09	3.74	1.98	0.007
Vitamin E, mg	9.20	5.55	8.20	4.08	6.99	3.14	<0.001
Iron, mg	13.06	5.35	12.37	4.39	11.63	4.09	0.001
Zinc, mg	9.13	3.80	8.90	3.35	8.82	3.59	0.53
Selenium, µg	52.54	22.81	48.93	24.16	45.06	17.00	<0.001
Folic acid, µg	306.96	128.60	273.79	95.07	250.20	86.47	<0.001
Beta-carotene, µg	4171.05	3973.60	2569.19	1338.44	1844.46	925.39	<0.001
Flavonols, mg	41.12	22.55	39.57	25.43	32.62	27.25	<0.001
Flavonones, mg	34.68	28.40	25.72	24.73	15.56	19.31	<0.001
Flavones, mg	0.33	0.30	0.29	0.19	0.26	0.16	<0.001
Tea, g	2.66	1.86	2.71	2.12	2.25	2.20	0.01
Caffeine, g	183.40	102.63	190.65	109.92	176.93	97.96	0.26

Continuous variables are presented as mean values and standard deviations; E-DII, energy-adjusted dietary inflammatory index; KJ, kilojoules; PUFA, polyunsaturated fatty acids; Higher E-DII scores indicate a pro-inflammatory diet; P values obtained using ANOVA.

Table 2 shows characteristics of the study participants at both time-points (age 70 and age 73) across the E-DII tertiles. The E-DII ranged from −3.82 to 5.01, with a mean of 0.59 (sd 1.63) which is slightly pro-inflammatory. Overall, participants in the highest tertile of E-DII scores (i.e., those with a pro-inflammatory diet) were more likely to be male, current smokers, less physically active, and less likely to have a history of hypercholesterolaemia, compared to those in tertiles 1 and 2. E-DII scores were not associated with chronic disease outcomes with the exception of hypercholesterolaemia (P = 0.046), such that a history of high cholesterol was associated with a lower E-DII (indicative of an anti-inflammatory diet).

**Table 2:** Participant characteristics across the tertiles of the energy-adjusted Dietary Inflammatory Index (E-DII) at age 70 (wave 1) and age 73 (wave 2) (Mean scores, or number of participants and percentages)

		**Tertiles of Dietary Inflammatory Index (E-DII)**						
	**Age 70 (n 928)**				**Age 73 (n 765)**			
	**Tertile 1 n 309**	**Tertile 2 n 310**	**Tertile 3 n 309**		**Tertile 1 n 256**	**Tertile 2 n 255**	**Tertile 3 n 254**	
**Characteristics**	**m or n(%)**	**m or n(%)**	**m or n(%)**	**P**	**m or (%)**	**m or (%)**	**m or (%)**	**P**
E-DII score	−0.13	0.64	2.37	<0.001				
Sex				<0.001				
Men	103 (33.3)	151 (48.7)	196 (63.4)					
Women	206 (66.7)	159 (51.3)	113 (36.6)					
Education (years)	10.81	10.83	10.74	0.64				
Age (years)	69.54	69.46	69.50	0.57	72.51	72.44	72.48	0.52
Depression (HADS-D)	2.51	2.79	2.87	0.10	2.29	2.63	2.72	0.06
CRP mg/l				0.03				0.29
Normal ≤3mg/l	174 (58.0)	156 (51.5)	140 (46.8)		151 (66.2)	131 (59.5)	134 (62.6)	
Elevated >3mg/l	126 (42.0)	147 (48.5)	159 (53.2)		77 (33.8)	89 (40.5)	80 (37.4)	
Fibrinogen, g/l	3.28	3.29	3.27	0.96	3.32	3.31	3.33	0.91
Normal ≤4.0g/l	262 (87.3)	251 (83.1)	258 (86.0)		203 (84.6)	211 (87.2)	207 (85.9)	
Elevated >4.0g/l	38 (12.6)	51 (16.9)	42 (14)		37 (15.4)	31 (12.8)	34 (14.1)	
hs-CRP mg/l†					1.79	2.06	2.17	0.09
Normal ≤3mg/l					189 (81.5)	176 (76.5)	171 (75.3)	
Elevated >3mg/l					43 (18.5)	54 (23.5)	56 (24.7)	
IL-6 pg/l†					1.82	1.90	2.21	0.02
Normal ≤1.6pg/l					143 (59.8)	126 (51.7)	114 (47.3)	
Elevated >1.6pg/l					96 (40.2)	114 (47.5)	127 (52.7)	
Physical activity (days/month)	8.37	7.76	6.68	0.03				
BMI kg/m2	27.44	27.73	27.64	0.68	27.68	27.65	27.67	1.00
Normal (<25)	88 (28.8)	74 (23.9)	73 (23.9)		60 (23.7)	59 (23.4)	68 (27.0)	
Overweight (25 to <30)	147 (48.0)	154 (49.8)	146 (47.9)		136 (53.8)	128 (50.8)	111 (44.0)	
Obese (≥30)	71 (23.2)	81 (26.2)	86 (28.2)		57 (22.5)	65 (27.8)	73 (29.0)	
Smoking status,				<0.001				<0.001
non-smoker	157 (50.8)	148 (47.7)	130 (42.1)		130 (50.8)	126 (49.4)	115 (45.3)	
ex-smoker	138 (44.7)	141 (45.5)	116 (37.5)		117 (45.7)	116 (45.5)	99 (39.0)	
current smoker	14 (4.5)	21 (6.8)	63 (20.4)		9 (3.5)	13 (5.1)	40 (15.7)	
Hypertension, n (%)				0.83				0.30
Yes	126 (40.8)	121 (39.0)	119 (38.5)		126 (49.2)	116 (45.5)	133 (52.4)	
No	183 (59.2)	189 (61.0)	190 (61.5)		130 (50.8)	139 (54.5)	121 (47.6)	
CVD, n (%)				0.27				0.62
Yes	73 (23.6)	65 (21.0)	82 (26.5)		74 (28.9)	66 (25.9)	75 (29.5)	
No	236 (76.4)	245 (79.0)	227 (73.5)		182 (71.1)	189 (74.1)	179 (70.5)	
Hypercholesterolaemia, n (%)				0.046				0.05
Yes	125 (40.5)	106 (34.3)	96 (31.1)		119 (46.5)	106 (41.6)	91 (35.8)	
No	184 (59.5)	203 (65.7)	213 (68.9)		137 (53.5)	149 (58.4)	163 (64.2)	
Stroke, n (%)				0.84				0.94
Yes	13 (4.2)	15 (4.8)	12 (3.9)		16 (6.2)	17 (6.7)	15 (5.9)	
No	296 (95.8)	295 (95.2)	297 (96.1)		240 (93.7)	238 (93.3)	239 (94.1)	
Diabetes, n (%)				0.58				0.99
Yes	24 (7.8)	27 (8.7)	20 (6.5)		25 (9.8)	24 (9.4)	25 (9.8)	
No	285 (92.2)	283 (91.3)	289 (93.5)		231 (90.2)	231 (90.6)	229 (90.2)	

Continuous variables are presented as mean values (and P values) and categorical variables are presented as numbers (and percentages); DII, dietary inflammatory index; HADS-D, Hospital Anxiety and Depression Score-Depression sub-test; MMSE, Mini-mental State Examination; CRP, C-reactive protein; IL-6, Interleukin-6; CVD, cardiovascular disease; fhs-CRP and IL-6 data were unavailable at age 70; P-values obtained using X2 tests or ANOVA tests, as appropriate.

Men and women did not differ in their concentrations of inflammation biomarkers. Elevated CRP concentrations (>3 mg/l) were found in 47.9% (n 432) of participants at wave 1 (age 70) and 37.2% (n 246) of participants at wave 2 (age 73); the correlation between non-hs-CRP at both time-points was r = 0.27 (P < 0.001). Elevated fibrinogen concentrations (> 4.0g/l) were found in 14.5% (n 131) at age 70 and 14.1% (n 102) at age 73; the correlation between fibrinogen at both time-points was r = 0.50 (P < 0.001). Elevated hs-CRP was found in 22.2% (n 153) of participants when measured a wave 2 (age 73). At age 73, the correlation between CRP and hs-CRP at age 73 was r = 0.80 (P < 0.001). Elevated IL-6 concentrations (> 1.6pg/l) were found in 46.8% (n 337) of participants at age 73. The correlation between hs-CRP and IL-6 at age 73 was r = 0.43 (P < 0.001).

Table 3 presents the results of multivariable logistic regression models. In the fully adjusted model, higher E-DII scores were significantly associated with higher CRP concentrations measured at age 70 (OR; 1.12, 95% CI 1.02, 1.24, P = 0.02) and higher IL-6 concentrations at age 73 (OR; 1.12, 95% CI 1.0042, 1.23, P = 0.04). No significant associations were observed between the E-DII and fibrinogen.

**Table 3:** Associations between the Dietary Inflammatory Index (E-DII) scores at baseline (age 70) and inflammation biomarkers as categorical variables at baseline and 3-year follow-up (ORs, and 95% confidence intervals)

		**Model 1**			**Model 2**	
**Biomarker**	**OR**	**95% CI**	**P**	**OR**	**95% CI**	**P**
Age 70						
Fibrinogen (>4g/l)	1.000	0.999, 1.001	0.70	0.983	0.870, 1.110	0.78
CRP (>3mg/l)	1.148	1.049, 1.257	0.003	1.122	1.018, 1.237	0.02
Age 73						
Fibrinogen (>4g/l)	0.953	0.833, 1.087	0.46	1.111	0.967, 1.276	0.14
CRP (>3mg/l)	1.066	0.964, 1.180	0.21	0.943	0.847, 1.050	0.28
hs-CRP (>3mg/l)	1.082	0.965, 1.213	0.18	0.964	0.852, 1.091	0.56
IL-6 (>1.6pg/l)	1.131	1.031, 1.242	0.009	1.112	1.004, 1.231	0.04

CRP, C-reactive protein; hs-CRP, high-sensitivity C-reactive protein; IL-6, interleukin-6; Model 1: adjusted for age, sex; Model 2: adjusted for age, sex, BMI, smoking status, physical activity, hypercholesterolaemia.

Post-hoc analyses (data not presented) showed that the statistically significant associations between E-DII scores and elevated CRP (age 70) and IL-6 (age 73) were strongest when comparing the highest tertile of the E-DII with the lowest (reference group, tertile 1). The odds of having an elevated CRP value (>3mg/l) for those in the highest E-DII tertile was 1.63 (95% CI 1.14, 2.35, P_trend_ = 0.008) times greater compared to those in lowest E-DII tertile. The odds of having an elevated IL-6 value (>1.6 pg/l) for those in the highest tertile of the E-DII was 1.60 (95% CI 1.11, 2.33, P_trend_ = 0.01) times greater than those in the lowest E-DII tertile.

## Discussion

This study found that higher E-DII scores were significantly associated with increased odds of having elevated plasma CRP and IL-6 among a sample of Scottish men and women in their seventies. Validation provides support for using the DII to predict systemic inflammation in later life. Our results suggest that a pro-inflammatory diet (high in cholesterol and saturated fats), and relatively lacking in anti-inflammatory foods (fruits and vegetables), is associated with significantly higher concentrations of two inflammatory biomarkers.

To the best of our knowledge, this is the first time that the DII has been validated in a sample of UK-based, and exclusively older, individuals. Previously, the DII has been validated in several US (16, 17, 19–21) and European (18, 22) populations. We observed significant associations when CRP was examined contemporaneously with E-DII at age 70, yet associations between baseline (age 70) E-DII scores and CRP and hs-CRP at age 73, were absent. Longitudinal studies of the DII are lacking. However, in the SU.VI.MAX cohort in France, the DII was not associated with CRP at a long-term (12-year) follow-up (22). Although statistical significance was not reached, associations between the E-DII and hs-CRP (CRP using the high-sensitivity procedure) in the present study, were linear and in the expected direction (with slightly smaller effect sizes). Comparable findings of non-significant associations have been reported in other studies investigating DII in relation to hs-CRP (17, 51). Associations between E-DII and fibrinogen were non-significant at both time-points. Unlike CRP and IL-6, there was no linear association between fibrinogen concentrations and E-DII score. Furthermore, there was little variance in plasma fibrinogen concentrations at each wave of testing, and little variation in change over time, limiting the ability to detect meaningful associations. However, some research indicates that hs-CRP and fibrinogen are less sensitive indicators of prevalent CVD, and CVD risk, than IL-6 (52).

Overall, our results are concordant with those of the Asklepios study (17) which found significant associations with IL-6 but no association between E-DII and hs-CRP or fibrinogen. Of the various markers of systemic inflammation, it appears that IL-6 is particularly relevant to aging. A review by Singh & Newman concludes that, of the inflammatory markers examined in cohort studies of aging, IL-6 is most robustly related to multiple disease outcomes, including CVD, diabetes, cancer, as well as disability and mortality (37). Mirroring this, clinical investigations have moved ‘upstream’ in the inflammatory cascade from CRP to IL-6 (and IL-1). There is increasing evidence that IL-6 ‘drives’ downstream inflammatory markers such as CRP and fibrinogen (53, 54), and as such, interleukins have been identified as more promising targets for intervention in atheroprotection (55).

This study also found that higher E-DII scores were associated with a range of unhealthy aging phenotypes. Those participants with a higher E-DII were more likely to be current smokers and less physically active. Smoking status was significantly and strongly associated with E-DII at both time-points and had a marked attenuating effect in the models. Chronic smoking induces the release of pro-inflammatory markers including IL-6 and acute-phase proteins and decreases the production of anti-inflammatory cytokines (56). Though physical exercise causes an acute-phase elevation of IL-6, in the long-term, regular physical activity has an anti-inflammatory effect via multiple pathways including cytokine production, improved endothelial function, and insulin sensitivity (57). Several other associations; e.g., with BMI, education and depression scores, were in the expected direction, in line with similar research, though not statistically significant. Thus, higher E-DII scores were found among those with a higher BMI, less education, and more symptoms of depression. The lack of a statistically significant association with BMI in this study is in contrast to many others which have examined DII in relation to BMI or obesity (e.g. 15, 58). There are two plausible explanations for this finding. First, by using BMI, we are examining the effect of overall obesity. However, other findings suggest that abdominal or ‘central’ obesity (in both obese and non-obese adults) is a specific contributor to inflammation (59, 60). Second, the predictive ability of BMI changes over time. Midlife obesity (related to the emergence of conditions such as hypertension) is related to higher risk of CVD, dementia and other outcomes, whereas it is not at older ages (61, 62). In fact, high BMI in later life (over the age of 65 years) becomes a predictor of decreased morbidity and mortality (63, 64).

The E-DII in our sample was not related to conditions such as CVD, stroke, diabetes or hypertension, despite the vast literature linking the DII with these conditions and their risk factors (see 65). Interestingly, a history of hypercholesterolaemia was associated with having lower E-DII scores. It is plausible that dietary modification, a first-line treatment strategy for elevated cholesterol levels, has lowered the inflammatory potential of the diet in those affected. Short-term improvements in DII scores have previously been observed among a sample of overweight and obese adults, in those randomised to plant-based diets, relative to those in the control (omnivore) group (66).

Overall, our study results are consistent with the hypothesis that diet modulates inflammation. Food items that tend to increase E-DII scores include butter, other animal fats, and high-fat sweets. Higher scores also could result from low consumption of food items considered to be anti-inflammatory such as fruits, vegetables and juices (18). Dietary characteristics unique to an older generation in Scotland, such as a higher intake of meat and saturated fat, and low intake of fresh produce (67), may explain some differences with other studies. Scotland’s unhealthy diet is widely cited as a factor in its’ poor health record (68). Of course, food choices in later life may be affected by illness, dental problems, gastrointestinal disorders and other aging issues. Although there are issues inherent in examining diet in an older population, our results suggest that the DII is a useful instrument for predicting inflammation biomarkers in later life.

This was the first study to validate the DII in a UK population, and in an exclusively older sample. Therefore, it has an important place in a growing area of academic enquiry. This manuscript also serves as a stepping stone for future papers in the same cohort. One of the strengths of the present study is the use of a validated and reproducible FFQ, which allowed for a comprehensive assessment of major nutrient sources in the diet. Other strengths include the availability of a range of covariates and two waves of data.

Limitations of this study include the non-availability of IL-6 and a high-sensitivity measure of CRP at baseline. Some measurement error inherent in the FFQ may be present; the list of foods is finite, portion sizes are estimates only, and self-reports may be biased. Although validated, the SCG-FFQ did not allow for the inclusion of all 45 food parameters required for optimal calculation of the DII. However, despite the reduction in the number of parameters, we still were able to successfully observe associations with some of the inflammatory markers, as have previous validation studies which have used fewer, e.g., the Asklepios study (17). It is possible that multiple testing may have resulted in chance associations being declared significant. In view of this, further replication is required. The DII score, calculated here from 26 components, ranged from −3.82 to 5.01. The lower (less pro-inflammatory) end of the continuum was less well represented in the LBC1936 than in other studies (e.g., 16, 19). Changes in the inflammatory potential of diet over time were not accounted for because only baseline dietary data were used to calculate the E-DII. There are many factors which determine serum concentrations of CRP, IL-6, and other cytokines, such as medical conditions and medications. We modelled the data on all the available potential confounders, but acknowledge the non-availability of medications data in the current study, including the pharmacological treatment of inflammation.

A lower percentage of participants in the elevated CRP group at age 73, compared with age 70, could reflect a healthy survivor effect in this aging cohort. Those with more pro-inflammatory diets, and higher levels of inflammation biomarkers, may suffer from health consequences in later life that preclude them from taking part in the study. Given that our sample comprised relatively healthy volunteers from an affluent area of Scotland, the findings may have limited generalisability. However, the most likely result of this, is a modest underestimation of the effect sizes of the associations present.

## Conclusions

In this study, E-DII scores were positively associated with CRP and IL-6 concentrations among an older-adult sample in Scotland. Validation of the E-DII further supports its use in assessing the potential impact of diet on inflammatory processes and extends the generalisability of this tool to an older (and British) population. Confirmation of these findings is warranted in order to further substantiate the utility of the DII to help in informing dietary intervention among an older population at risk of chronic disease.

*Financial support:* The present study was funded by the Age UK’s Disconnected Mind project. The work was undertaken in the University of Edinburgh Centre for Cognitive Ageing and Cognitive Epidemiology, part of the cross-council Lifelong Health and Wellbeing Initiative, which is funded by the Medical Research Council Biotechnology and Biological Sciences Research Council (MR/K026992/1). Drs. Shivappa and Hébert were supported by grant number R44DK103377 to CHI from the United States National Institute of Diabetes and Digestive and Kidney Diseases.

*Ethics statement:* The study was conducted according to the guidelines laid down in the Declaration of Helsinki, and all procedures were approved by the Multi-Centre Research Ethics Committee for Scotland (MREC/01/0/56) and from the Lothian Research Ethics Committee for Scotland (LREC/2003/2/29). Written informed consent was obtained from all participants.

*Conflict of interest:* J. R. H. owns controlling interest in Connecting Health Innovations LLC (CHI), a company that has licensed the right to his invention of the dietary inflammatory index (DII) from the University of South Carolina in order to develop computer and smart phone applications for patient counseling and dietary intervention in clinical settings. N. S. is an employee of CHI. None of the other authors declares any conflicts of interest.

## Abbreviations

CRP: C-reactive protein
DII: dietary inflammatory index
hs-CRP: high-sensitivity C-reactive protein
